# First third-generation CAR T cell application targeting CD19 for the treatment of systemic IgM AL amyloidosis with underlying marginal zone lymphoma

**DOI:** 10.1186/s40364-023-00532-2

**Published:** 2023-10-14

**Authors:** Felix Korell, Stefan Schönland, Anita Schmitt, Madelaine Jansen, Kiavasch Farid, Carsten Müller-Tidow, Peter Dreger, Michael Schmitt, Ute Hegenbart

**Affiliations:** Department of Hematology & Oncology, Amyloidosis Center, University Hospital Heidelberg, Im Neuenheimer Feld 410, 69120 Heidelberg, Germany

**Keywords:** CAR T cell therapy, Amyloidosis, Free light chains, IgM, Marginal zone lymphoma

## Abstract

Light chain amyloidosis (AL) is a rare disease caused by the generalized deposition of misfolded free light chains. Patients with immunoglobulin M gammopathy (IgM) and indolent B-cell lymphoma such as marginal zone lymphoma (MZL) may in some instances develop AL amyloidosis. So far, CAR T cells for AL amyloidosis have only been reported utilizing the B cell maturation antigen as target, while CD19 has so far not been used in AL amyloidosis.

We report the case of a 71-year-old male, diagnosed with systemic AL kappa amyloidosis and MZL, receiving third-generation CAR T cell therapy targeting CD19. Prior treatment included bendamustine/rituximab and cyclophosphamide/ dexamethasone with subsequent autologous stem cell transplantation. CAR T application was well tolerated despite heart and kidney amyloid manifestations, and only early low-grade procedure-specific toxicities were observed. A continuous decrease in IgM, kappa light chains and kappa-to-lambda light chain difference was observed in the patient from day + 30 on, resulting in a deep hematological response six months after treatment.

In summary, we present a novel case of CAR T cell treatment with third generation CD19 directed infusion for AL amyloidosis with an underlying secretory active B cell lymphoma, showing that this is an effective treatment modality and can be applied to patients with subsequent AL amyloidosis.

To the Editor,

Amyloid light chain amyloidosis (AL amyloidosis) is a rare protein deposition disorder that results in potentially serious organ dysfunction and remains an uncurable disease [1]. In most cases, the underlying disease is a clonal plasma cell disease that produces excess amyloidogenic light chains and in rare instances, patients with immunoglobulin M (IgM) gammopathy and indolent B cell lymphoma like marginal zone lymphoma (MZL) and Waldenstrom's macroglobulinemia may also develop AL amyloidosis [2].

Meanwhile, chimeric antigen receptor (CAR) T cells have emerged as a successful pillar of therapy in the treatment of hematological malignancies. While prior two single-center attempts in AL amyloidosis patients have focused on CAR T cells against the B cell maturation antigen [3, 4], mostly expressed by plasma cells, targeting CD19 could offer a therapeutic option for patients with AL amyloidosis and secretory active indolent B cell lymphoma.

Here we report a first case of systemic AL kappa amyloidosis and MZL treated with our academic third-generation CAR T cell therapy targeting CD19.

Our patient was a 71-year-old white male with an asymptomatic MZL stage IVa (diagnosed with a lymph node biopsy) and subsequent AL amyloidosis (diagnosed two years later with a renal biopsy while becoming nephrotic), with kidney (stage II [5]) and heart (stage IIIa [6]) manifestation (for details Table 1). He received lymphodepletion chemotherapy followed by a single-dose CAR T cell infusion as 3^rd^-line treatment. Prior to CAR T cell consideration, the patient had received two different treatment regiments with regards to his lymphoma disease, beginning initial treatment with bendamustine/rituximab, and as second line cyclophosphamide/ dexamethasone with subsequent autologous stem cell transplantation. After progressing 33 months later, bridging therapy was applied with bortezomib/rituximab with no response before leukapheresis for CAR T cell manufacturing.

**Table 1:** Clinical and laboratory evaluation, including treatment lines

**Month/Year**	**Clinical course (diagnosis / progress / treatment)**	**Timepoint laboratory parameters:**	**IgM** **(g/L)**	**dFLC (mg/L)**	**Best achieved hematologic/organ response**	**NT-proBNP (ng/L)**	**Creatinine (mg/dL)**	**Proteinuria (g/day)**
Dec 2016	initial AL diagnosis	At diagnosis (Dec 2016)	12.3	79.5	-	1700 NYHA II	1.07	5.177
Dec 2016	treatment with **R-Bendamustine**	After treatment (May 2017)	3.03	27.9	VGPR	2642	1.03	3.209
Nov 2018	progress	At progress (Nov 2018)	4.46	68.8	-	2295	1.20	0.802
Nov 2018	mobilisation chemotherapy (**cyclophosphamide/dexamethasone**) with stem cell collection	After treatment (Dec 2018)	3.47	66.6	Renal response	3129	1.11	0.324
Jan 2019	consolidation—**autologous stem cell transplantation** conditioned with HD **melphalan** (200mg/m^2^)	After treatment (Apr 2019)	1.31	11.1	VGPR	1859	1.24	0.273
Oct 2021	progress	At progress (Oct 2021)	6.36	149.6	-	2109	1.34	0.148
Apr 2022	bridging with **R-Bortezomib**	After bridging (Jun 2022)	4.74	127.1	non-response	1522	1.30	0.168
Aug 2022	*prior admission for CAR T cell infusion*	*Prior CAR T cell infusion (Aug 2022)*	*3.33*	*105.4*	*-*	*1916*	*1.20*	*0.156*
Aug 2022	lymphodepleting chemotherapy (**cyclophosphamide** 500mg/m^2^/day and **fludarabine** 30mg/m^2^/day on days -4, -3 and -2) and **CAR T cell infusion**	After CAR T cell infusion (Sept 2022)	1.83	83.6	non-response	1868	1.84	-
Nov 2022	3-month CAR T cell follow-up	3-month follow-up (Nov 2022)	0.99	54.6	PR	2047	1.43	-
Feb 2023	6-month CAR T cell follow-up	6-month follow-up (Feb 2023)	0.91	30.2	VGPR	2544	1.23	0.171

*R* Rituximab, *HD* high dose, *CAR* chimeric antigen receptor, *IgM* immunoglobulin M, *dFLC* difference between involved (kappa) and uninvolved (lambda) free light chains, *NT-proBNP* N-terminal brain natriuretic peptide, *VGPR* very good partial response

Due to the low toxicity induced by our third-generation product [7] and to not impair efficacy, no changes to the procedure were made; and the patient, after consultation with colleagues from our amyloid center, was cleared for treatment.

CD19-CAR Ts reached peak expansion at day + 7 with 346,414 copies per µg peripheral blood mononuclear cells (PBMC) DNA assessed by single copy gene duplex quantitative PCR (Fig. 1A), displaying high persistence with > 20,000 copies measured at days + 43 to + 84. The CAR T cell therapy was well tolerated and displayed low grade procedure-specific toxicity with only grade 1 immune effector cell-associated neurotoxicity syndrome (ICANS). Fever during aplasia resolved under antibiotic therapy with initially piperacillin/tazobactam and subsequent escalation to meropenem; with only slightly elevated interleukin 6, ferritin and C reactive protein levels (Fig. 1B). The patient received antibacterial (rifaximin), antiviral (acyclovir) and antifungal (fluconazole) prophylaxis for a month after discharge. Cardiac biomarker returned to pre-infusion values outside of a late N-terminal brain natriuretic peptide rise to 2,544 ng/L at d + 171 (Fig. 1C). No electrocardiogram changes were seen after CAR T cell infusion, with the patient maintaining a priorly diagnosed atrial fibrillation. Most parameters stayed consistent or achieved the pre-infusion level at the later FU, with left ventricle end diastolic and end systolic diameters (LV-EDD and LV-ESD, respectively) as well as tricuspid annular plane systolic excursion (TAPSE) increasing. Renal functional markers as well as liver parameters all returned to pre-treatment levels (Fig. 1D).

**Fig. 1:**
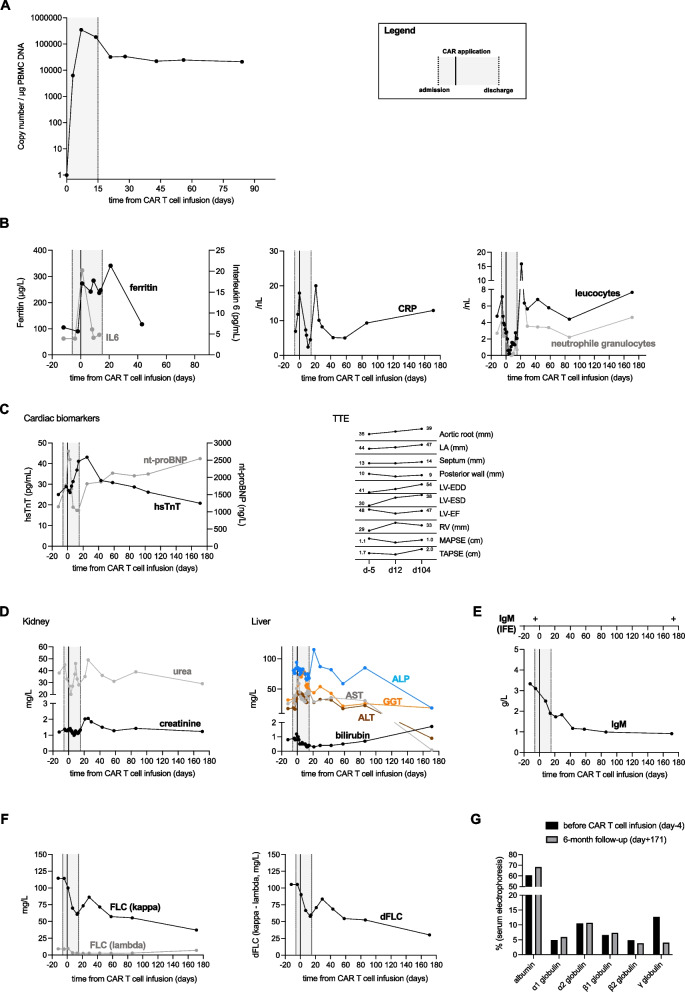
Overview of treatment- and disease-related parameters

From day + 30 after CAR infusion onwards, the patient experienced a continuous drop of IgM, kappa light chains and kappa-to-lambda light chain difference due to persisting CAR T cell activity (Fig. 1E and F), achieving a deep hematologic response graded as very good partial remission at six months after treatment [8]. Additionally, the gamma-globulin fraction in serum electrophoresis was reduced from 12.7 (before CAR infusion) to 4% (Fig. 1G), with no detectable M-gradient (and in fact the lowest IgM level since diagnosis), however immunofixation stayed positive for IgM kappa (Fig. 1E). This is a direct proof that choosing CD19 as target is appropriate to treat both the FLC producing more plasmacytic differentiated clone) and the IgM producing clon) and strongly suggests third generation CD19 CAR activity towards MZL.

Unfortunately, shortly after the 6-month evaluation, the patient suffered a severe respiratory infection and required hospitalization in a local hospital. Infections are a common complication after CD19 CAR T cells, occurring in 18–60% of patients when assessing approval trial and real-world data, and even 30 days and later after CAR infusion [9]. The condition subsequently worsened, with the patients developing a sepsis caused by *haemophilus influenzae*. Despite intensive care measures, the patient died of multiorgan failure on day + 195 after CAR T cell infusion.

Limiting the general evaluation of CAR T cell application in this setting is the death of the patient due to infectious complications after six months, preventing long-term response analysis on organ responses. At day + 180, there was no improvement of cardiac and renal biomarkers yet.

In conclusion this case suggests that CD19-directed CAR T cell therapy for MZL with systemic AL amyloidosis is feasible and clinically potent by effectively attacking the IgM and FLC secreting B cell clones, leading to a significant reduction in IgM and amyloidogenic kappa-FLCs levels. Patient selection and monitoring during and after therapy are important cornerstones in this high-risk patient cohort.

## Data Availability

All data and materials can be made available by the authors upon reasonable request.
